# Spontaneous pneumomediastinum, pneumothorax and subcutaneous emphysema in COVID-19 patients—a case series

**DOI:** 10.1186/s43055-020-00401-0

**Published:** 2021-01-14

**Authors:** Akshat Agrawal, Kamal Kumar Sen, Gitanjali Satapathy, Humsheer Singh Sethi, Ajay Sharawat, Dwarampudi Sindhu Reddy

**Affiliations:** Department of Radiodiagnosis, Kalinga Institute of Medical Sciences, Bhubaneswar, India

**Keywords:** Spontaneous pneumomediastinum, Pneumothorax, Subcutaneous emphysema, COVID-19

## Abstract

**Background:**

Spontaneous pneumomediastinum, pneumothorax and spontaneous subcutaneous emphysema are rare entities. A rising trend in the setting of COVID-19 even in patients who are not put on invasive ventilation can suggest an alternative aetiology.

**Case presentation:**

We describe four cases which presented with suspected symptoms of COVID-19 and were diagnosed with pneumomediastinum, pneumothorax, and subcutaneous emphysema which would have been missed if not for computed tomography scan performed at the time of admission. Three of these cases had no prior history of any iatrogenic intervention, and the fourth person developing pneumothorax and subcutaneous emphysema after intubation.

**Conclusions:**

Pneumomediastinum, pneumothorax and subcutaneous emphysema can be noted as a complication of COVID-19 itself as well as the complication of management of COVID-19.

## Background

The first case of COVID-19 in India was reported on January 30, 2020, and has since shown a peak with current numbers standing at 5.7 million cases and 91,149 deaths [1]. While subcutaneous emphysema and spontaneous pneumomediastinum have been observed in patients with a variety of viral pneumonia as a complication of mechanical ventilation, the development of these conditions in non-intubated patients suggests an alternative aetiology [2]. Over the last few months, we have noted an increase in the patients presenting with pneumomediastinum and subcutaneous emphysema, with confirmed COVID-19 status more so in those who were intubated, raising the question if it is more so because of the viral disease or the complication of the emergent procedure.

## Case presentation

### Case 1

**Fig. 1:**
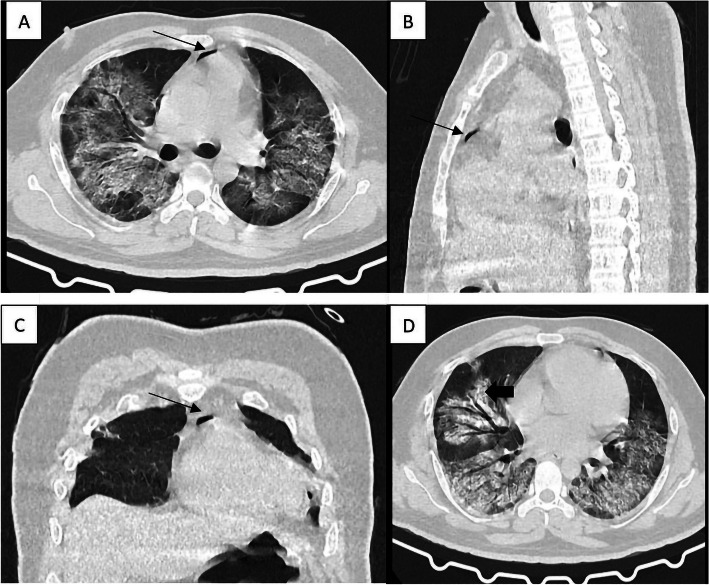
Axial images (**a**, **d**), sagittal reconstruction (**b**) and coronal reconstruction (**c**) showing the presence of gas in the mediastinum (black arrows) and diffuse parenchymal consolidation with air bronchogram in bilateral lung fields (block arrow)

A 35-year-old male presented with cough for 7 days and mild shortness of breath for the last 5 days with a history of contact to a known case of COVID-19 before developing symptoms. Reverse transcriptase-polymerase chain reaction (RT-PCR) was positive for COVID-19. Day 1 chest x-ray and high-resolution computed tomography scan (HRCT) revealed diffuse ground-glass opacities (GGO) with interlobular septal thickening in bilateral lung fields. Repeat x-ray on day 10 revealed the development of pneumothorax which was drained subsequently with an intercostal drainage tube. CT was repeated on day 14 following the worsening of dyspnea which revealed a small area of pneumomediastinum (Fig. 1) following which he was intubated and put on mechanical ventilation. On day 18, the patient developed sudden bradycardia and hypotension for which inotropic support was provided and resuscitation performed but patient succumbed and was declared dead.

### Case 2

**Fig. 2:**
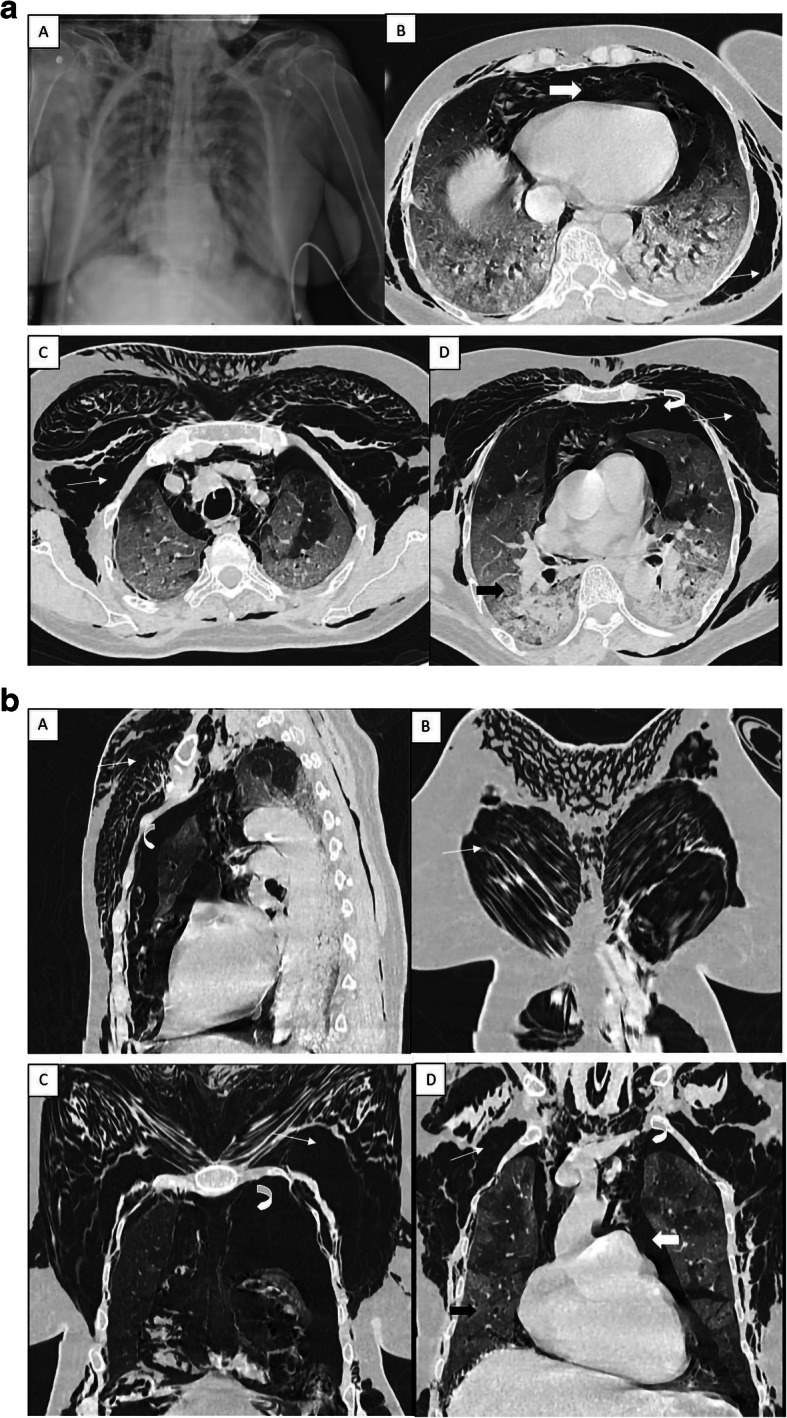
**a** Chest x-ray (A) demonstrating patchy opacities of consolidation, subcutaneous emphysema in the bilateral cervical region and chest wall along with a thin stripe of air along trachea and mediastinum. Axial HRCT images (B, C, D) demonstrating extensive subcutaneous emphysema (white arrows) in the bilateral chest wall, pneumothorax with partial collapse of bilateral lungs (curved arrow) and pneumomediastinum (block white arrow). Lung parenchyma shows diffuse ground-glass opacities with scattered consolidation in posterior segments (black block arrow). **b** Sagittal (A) and coronal reconstruction (B, C, D) of the same patient showing extensive subcutaneous emphysema (white arrows) in the bilateral chest wall, pneumothorax with partial collapse of bilateral lungs (curved arrow) and pneumomediastinum (block white arrow). Bilateral lung parenchyma shows diffuse ground-glass opacities (black block arrow)

A 68-year-old diabetic female, presented with a history of cough for 7 days, severe breathlessness and palpitations for 1 day. RT-PCR was positive for COVID-19. HRCT chest done at admission revealed diffuse GGO, interlobular septal thickening in bilateral lung fields along with diffuse pneumomediastinum, bilateral pneumothorax and diffuse subcutaneous emphysema of bilateral chest wall (Fig. 2a, b). The patient was shifted to ICU (intensive care unit) where she was intubated and put on multiple ionotropic support. The patient developed features of multiorgan dysfunction and had an episode of seizure after which her condition worsened and was declared dead on day 8.

### Case 3

**Fig. 3:**
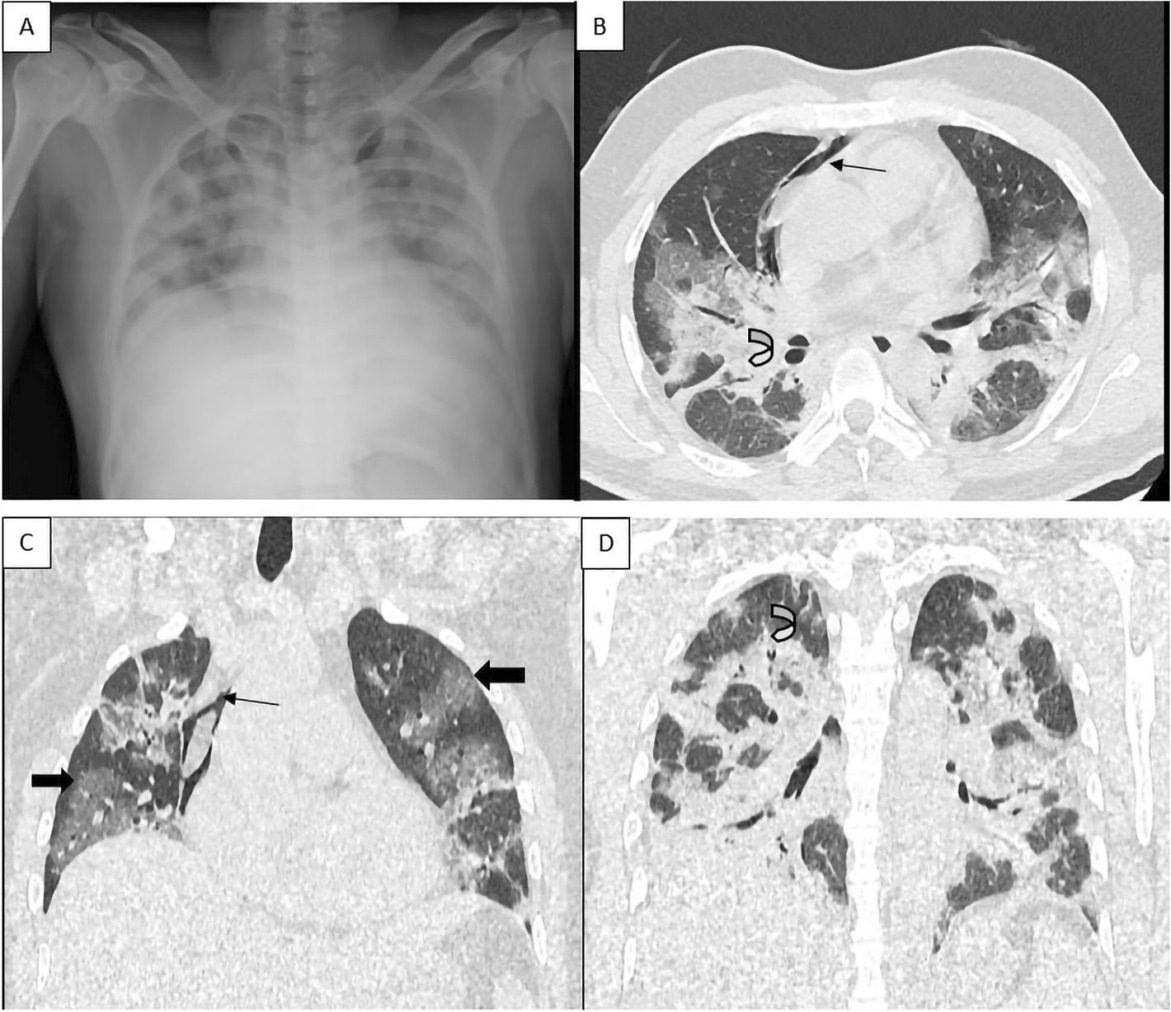
Chest x-ray (**a**) non-homogenous patchy opacities in bilateral lung fields suggestive of consolidation. Axial HRCT section (**b**), coronal sections (**c**, **d**) demonstrate the presence of air in the mediastinum (black arrow) along with the presence of diffuse ground-glass opacities (block arrow) and consolidation (curved arrow) with air bronchogram in the bilateral lung parenchyma

A 31-year-old male presented with fever of 5 days duration. At the time of admission, the patient had breathlessness along with hypoxia. The patient was a laboratory-proven case of COVID-19 referred from a primary health centre. HRCT chest revealed consolidation, GGO, septa thickening and atelectatic changes in bilateral lung fields with pneumomediastinum (Fig. 3). The patient was shifted to ICU due to worsening of dyspnea and was managed with oxygen inhalation and other medications. The patient improved over the period and was discharged in a hemodynamically stable condition on day 25.

### Case 4

**Fig. 4:**
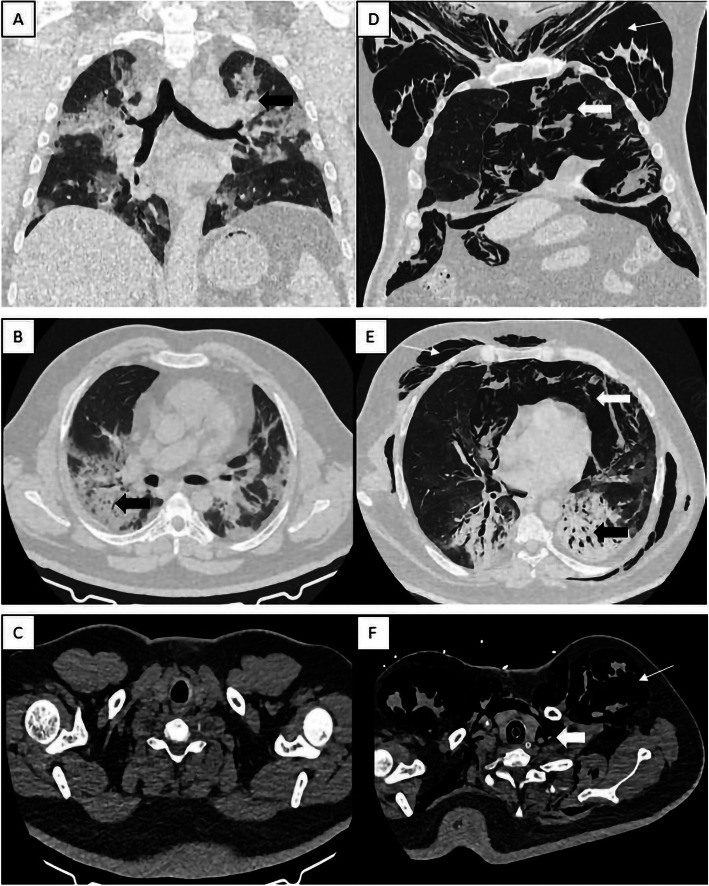
Pre (**a**, **b**, **c**) and post (**d**, **e**, **f**) intubation HRCT chest images demonstrating the presence of consolidation (black block arrow) in bilateral lung fields. Presence of diffuse subcutaneous emphysema (white arrow) is noted along bilateral chest wall extending into arms and back along with pneumomediastinum (white block arrow) extending up to the paratracheal area

A 37-year-old paediatrician presented with fever of 5-day duration, breathlessness and cough for 2 days. RT-PCR was positive for COVID-19. HRCT chest done at admission revealed diffuse areas of GGO and consolidation in bilateral lung fields with a CT severity score (CTSI) of 13/25. The patient was put on non-invasive ventilation because of him desaturating on room air and was finally intubated on day 10. Repeat CT on day 20 revealed massive subcutaneous emphysema along with pneumomediastinum and pneumoperitoneum (Fig. 4) in addition to aggravation of further chest findings and a CTSI of 22/25. The patient was put on extracorporeal membrane oxygenation (ECMO) and since then has been showing positive results. The patient at the time of writing this article is in a stable condition.

## Discussion

Our case series focusses on the development of spontaneous pneumomediastinum, pneumothorax or surgical emphysema in the three COVID-19 diagnosed cases with no previous history of intubation and one patient with a history of intubation developing subcutaneous emphysema post-intubation. Pneumomediastinum is most often caused by increased airway pressures, secondary to mechanical ventilation or airway obstruction; however, other causes include a rise in intrathoracic pressure (such as from the Valsalva manoeuvre); strenuous activity; severe vomiting (diabetic ketoacidosis, anorexia nervosa); trauma to the thoracic cavity; oesophageal rupture; thoracic and head and neck surgery, particularly with resultant tracheobronchial injury; and alveolar injury due to underlying diseases such as infection and sarcoidosis [3].

Contrary to the previous statement, we see that the findings revealed on chest CT was noted even before any iatrogenic intervention was performed which led us to believe that these severe conditions were sequelae of COVID-19 rather than being an adverse effect of mechanical/barotrauma. One of the most important points to be noted here is that none of the four patients had any previous history of respiratory disorder or smoking habit. There have been previous case reports citing similar data in the setting of COVID-19 [4, 5]. One of the possible mechanisms of injury involved in these cases could be a result of diffuse alveolar injury in severe COVID-19 disease, wherein the alveoli may be prone to rupture [5]. Two of our patients had a cough which could also be an additive factor in alveolar rupture. This may lead to spontaneous pneumomediastinum through Macklin’s phenomenon. Interstitial air can then dissect into the mediastinum, pleural cavity and subcutaneous tissues. Similar pathological progressions have been previously observed in a variety of viral pneumonia [6]. Macklin described how alveolar air which is released from alveolar rupture tracks along peribronchial vascular sheaths towards the mediastinum [7].

While searching for literature, a prior study showed that only about 1% of COVID-19 patient has pneumothorax [8]. In our centre, a study on 3500 patients revealed only 15 (0.43%) patients developing pneumothorax, pneumomediastinum or subcutaneous emphysema with intubation-related barotrauma being attributed as the aetiology to 12 cases (80%) while 20% cases were designated spontaneous, as a sequela of COVID-19. Rupture of emphysematous bulla could be one of the causes of the development of pneumothorax which subsequently could result in subcutaneous emphysema. The literature search revealed a case study in which the patient similarly had spontaneous pneumothorax, pneumomediastinum and surgical emphysema similar to case 2 in our series [9].

Dyspnea being a non-specific symptom could be present in moderate to severe COVID-19, pneumomediastinum and pneumothorax. All the four patients had dyspnea and three of four patients developing a cough and two of the three patients presenting with fever. All four patients needed mechanical ventilation to overcome the dyspnea. Two of the four patients had a fatal outcome. It is worthwhile to note that three of four patients belonged to a young age group.

The fourth patient in our series presented with all the usual symptoms of COVID-19 and showed GGO and consolidation in the initial CT scan without any signs of pneumomediastinum. It is only after the intubation that the patient developed pneumomediastinum and subcutaneous emphysema. COVID-19 is recognised as an aetiological factor for causing central and upper airway inflammation and oedema leaving patients potentially vulnerable to injury from instrumentation. Furthermore, expeditious intubation due to severe hypoxaemia in emergent settings could be a contributory factor to the tracheobronchial injury [10]. Subcutaneous emphysema is the most common finding in tracheal lacerations. It serves as the sentinel sign that stimulates further confirmatory studies to establish the diagnosis. Other signs include mediastinal emphysema, pneumothorax, dyspnea, dysphonia, cough, hemoptysis and pneumoperitoneum [11]. The process to reposition the patient to prone in heed to balance the ventilation-perfusion mismatch has certain risks of its own [12] and could have been one of the factors following which there is an increased chance of the injury of an already susceptible tracheobronchial tree.

Chest x-ray is the diagnostic standard for pneumomediastinum, half of all cases may be missed without a lateral film [13]. CT scan remains the definitive diagnostic tool. This will demonstrate subcutaneous emphysema, pneumopericardium and potential tracheobronchial injuries alongside the bilateral infiltrates typical of COVID-19 [14].

## Conclusion

Pneumomediastinum and pneumothorax are a not so common finding associated with COVID-19, can be seen as a poor prognosis for the patient and increased morbidity and prolonged hospital stay for the patients. Pneumomediastinum, pneumothorax and subcutaneous emphysema can be noted as a complication of COVID-19 itself as well as the complication of management of COVID-19. A susceptible trachea in combination with altered immune status, emergency intubation, frequent proning and high positive end-expiratory pressure (PEEP) can lead to an increase in the occurrence of pneumomediastinum and subcutaneous emphysema. Other factors including but not limited to large turnover of the COVID-19 patients, paucity of skilled health workers, long working hours and fear of infection amongst the medical fraternity can add up to the risk of complications. Regular interval follow-up with inflammatory marker levels and follow-up CT post-admission especially in a refractory case can prove to be a boon for the patient.

## Data Availability

The datasets used and/or analysed during the current series are available from the corresponding author on reasonable request.

## Abbreviations

RT-PCR: Reverse transcriptase-polymerase chain reaction
HRCT: High-resolution computed tomography scan
GGO: Ground-glass opacities
ICU: Intensive care unit
CTSI: CT severity index
ECMO: Extracorporeal membrane oxygenation
